# Long-term outcomes of concomitant tricuspid valve repair in patients undergoing mitral valve surgery

**DOI:** 10.1186/s13019-020-01244-6

**Published:** 2020-08-04

**Authors:** Ayse Cetinkaya, Natalia Ganchewa, Stefan Hein, Karin Bramlage, Peter Bramlage, Markus Schönburg, Manfred Richter

**Affiliations:** 1 Department of Cardiac Surgery, Kerckhoff-Heart Center Bad Nauheim, Campus of the University Hospital Giessen, Benekestraße 2-8, 61231 Bad Nauheim, Germany; 2 Department of Anesthesiology, Kerckhoff-Heart Center Bad Nauheim, Bad Nauheim, Germany; 3 Institute for Pharmacology and Preventive Medicine, Cloppenburg, Germany

**Keywords:** Mitral valve surgery, Tricuspid valve repair, Survival, Complications

## Abstract

**Background:**

We aimed to find out how the concomitant performance of tricuspid valve repair (TVR) affects outcomes of patients undergoing mitral valve surgery (MVS).

**Methods:**

Single-centre, retrospective analysis of 1357 patients who underwent MVS between January 2005 and December 2015, including 1165 patients with isolated MVS and 192 patients with MVS plus TVR. We used propensity scores to match patients for baseline characteristics other than valve related parameters and arrived at a matched sample of 182 patients per group.

**Results:**

The overall procedure duration was longer in the MVS + TVR (224 min) versus the MVS group (176 min; *p* < 0.001), as were the duration of mechanical ventilation (13 vs. 11 h; *p* < 0.001), X-clamp (90.5 vs. 66 min; *p* < 0.001) and cardiopulmonary bypass time (136 vs. 95.5 min; *p* < 0.001). Rates of procedural complications were not different between groups with the exception of pacemaker rates which were 16.0% in the MVS + TVR group and 8.8% in the isolated MVS group (*p* = 0.037).

There was no difference in death rates within 30 days, stroke, myocardial infarction or repeat MVS. The long-term survival rate was 60.8% in the MVS + TVR vs. 57.5% in the isolated MVS group (HR 1.048; 95%CI 0.737–1.492; *p* = 0.794). The rate of grade III/IV tricuspid regurgitation (TR) remained low after MVS + TVR during long-term follow-up while the rate of grade ≥ II TR increased slightly in the isolated MVS group.

**Conclusion:**

The data show that the concomitant performance of TVR in patients undergoing MVS is a safe and effective procedure with good long-term outcomes. Patients can undergo MVS + TVR with confidence as it improves their prognosis up to the level of patients undergoing isolated MVS.

## Introduction

Patients requiring mitral valve (MV) surgery (MVS) often suffer from concomitant tricuspid valve (TV) regurgitation (TR). Whether or not to manage concomitant TR at the time of mitral valve (MV) surgery (MVS) is highly controversial. As a result, the frequency of concomitant TV repair (TVR) during MVS ranges from 7 to 65% at different centres around the world [1]. The dispute is mostly over patients with mild or moderate TR with or without annular dilation.

Clinically it is a difficult situation to explain patients that they need to undergo concomitant TVR as the procedure usually takes longer and the potential increase in complications may exceed the benefit. While there is less dispute in TR grade III/IV [2, 3], this usually applies to moderate and also mild TR patients. Physicians who take a conservative approach would only intervene on the tricuspid valve in parallel to MVS in cases with severe TR or risk factors for progression of TR, because they usually expect that MVS will also restore tricuspid valve function in less than severe cases. Physicians who manage TVR more aggressively usually do so because of the increased mortality and morbidity associated with repeat surgery for TVR performed after MVS, and because concomitant TVR is generally a safe procedure [4, 5]. A recent meta-analysis of 17 studies compared TVR to no intervention during MVS, with a mean follow-up of 6.0 years. The authors found no difference in 30-day/in-hospital or late mortality between patients with or without TVR [6]. TVR protected against late moderate and severe TR. On the other hand, the need for permanent pacemaker implantation (PPI) was higher in patients who underwent TVR.

In an attempt to validate these results in our own patient population, we performed a retrospective analysis of our 1357 patients intervened between January 2005 and December 2015 at the Kerckhoff-Heart Center Bad Nauheim, Germany. We aimed to explore the impact of concomitant TVR at the time of MVS on procedural parameters, procedure-related and 30-day complications, and long-term survival and to compare it with the outcomes of isolated MVR.

## Materials and methods

This study was a single-centre, retrospective analysis of MVS [7]. Patients undergoing MVS at our site within the specific time period were included in the study. The analysis included patients who underwent isolated MVS or MVS combined with TVR (MVS + TVR). The study was approved by the site’s ethical committee and complied with the Declaration of Helsinki and its amendments. Given that the study used anonymised data already collected as part of routine diagnosis and treatment, written informed consent was not required.

### Data, outcomes and definitions

All electronic medical records for patients who had undergone MVS were reviewed (including inpatient and outpatient notes and the results of any diagnostic testing). Recorded clinical variables included patient age, sex, comorbid diseases, prior cardiology procedures, echocardiographic procedures and other pertinent medical/surgical history. Follow-up data concerning complications and echocardiographic parameters were collected at the patient’s last hospital follow-up visit.

### Statistics

Propensity score (PS) matching was performed to account for differences in patient characteristics at baseline other than the valve disease itself. The propensity score for each patient was calculated by logistic regression with adjustment for 12 key baseline variables, including age, gender, diabetes, renal insufficiency, atrial fibrillation, prior aortic valve replacement, prior coronary artery bypass grafting, New York Heart Association (NYHA) score ≥ 3, pulmonary hypertension, log EuroScore I, emergency indication, and left ventricular ejection fraction (LVEF). A difference in propensity score of 1% (0.01) was tolerated when matching patients 1:1.

Data were analysed using descriptive statistics, with categorical variables presented as absolute values and frequencies (%) and continuous variables presented as mean and standard deviation or median and interquartile range (IQR). Comparisons between the isolated MVS and MVS + TVR groups were carried out using a t-test or Mann-Whitney U test for continuous variables and a Fisher’s exact or Chi-square test for categorical variables. Survival analyses were presented as Kaplan-Meier curves. In addition, hazard ratios (HR) were calculated by Cox-regression.

In all cases, a two-tailed *p*-value of < 0.05 was considered statistically significant. All statistical tests were performed using IBM SPSS Statistics software version 24.0 (IBM Corporation, Armonk, New York, USA).

## Results

**Fig. 1:**
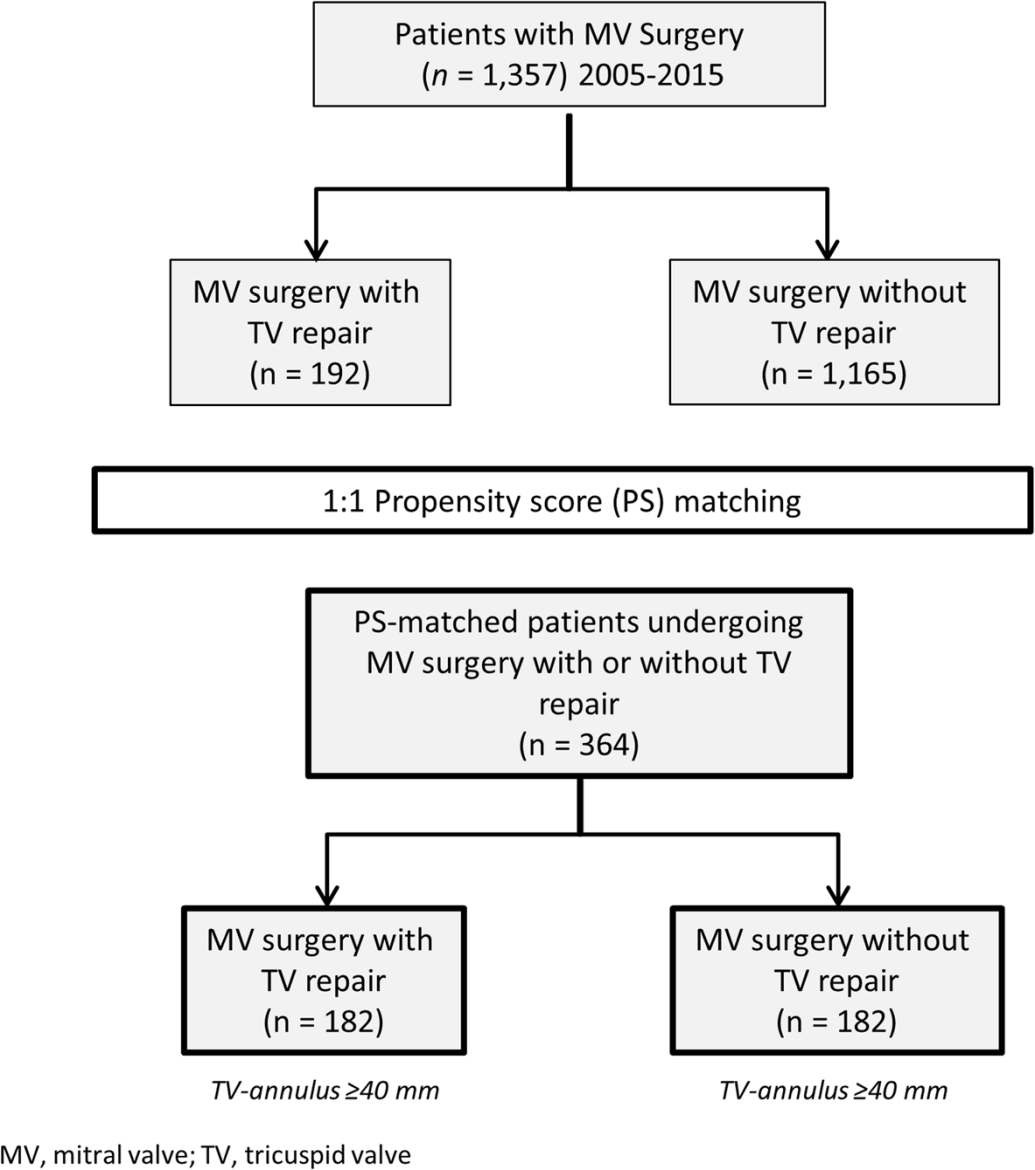
Flow chart of patient disposition

Our MV database comprised 1357 patients who underwent MVS in the indicated time period (Fig. 1). MVS + TVR was performed in 192 patients and isolated MVS in 1165 patients. Propensity score matching (as outlined above) resulted in 182 patients per group.

### Patient characteristics

In the overall (unmatched) MV population, patients had a mean age of 63.9 years and 43.3% were female; atrial fibrillation (32.4%) and pulmonary hypertension (12.0%) were frequent and potentially associated with the MV disease (Table 1). The majority of patients were highly symptomatic, with 75.5% being in NYHA class III or IV. Between-group differences for the overall (unmatched) population were abundant, but propensity score matching resulted in two comparable patient groups with some numerical but without any statistically significant difference between them (Table 1).

**Table 1:** Patient characteristics

	All patients documented				PS matched cohort		
	Total ***N*** = 1357	Isolated MVS ***N*** = 1165	MVS + TVR ***N*** = 192	***p***-value	Isolated MVS ***N*** = 182	MVS + TVR ***N*** = 182	***p***-value
Age (years)	63.9 ± 12.3	63.0 ± 12.5	69.2 ± 10.1	< 0.001	69.4 ± 10.9	68.9 ± 10.2	0.623
Female gender, %	43.3	40.9	58.3	< 0.001	57.7	57.7	1.000
Body mass index (kg/m^2^)	26.4 ± 4.7	26.4 ± 4.6	26.3 ± 5.1	0.807	26.1 ± 25.7	26.3 ± 5.1	0.716
Cardiovascular risk factors							
Hypertension, %	53.7	53.2	56.8	0.360	51.6	57.1	0.292
Dyslipidaemia, %	16.8	17.2	14.7	0.390	13.7	15.5	0.640
Diabetes mellitus, %	8.7	8.0	13.0	0.022	9.9	13.7	0.256
Comorbidities - general							
Creatinine (mg/dL)	1.0 ± 0.5	1.0 ± 0.4	1.1 ± 0.6	< 0.001	1.1 ± 0.4	1.1 ± 0.5	0.518
Kidney failure (Cr > 2.26 mg/dL), %	1.7	1.3	4.2	0.010	3.3	3.3	1.000
Stroke, %	5.5	5.3	6.3	0.600	4.9	6.0	0.645
COPD, %	11.8	11.7	12.5	0.742	11.0	12.6	0.626
PAD, %	3.0	2.7	4.7	0.145	4.4	4.4	1.000
Comorbidity – cardiac							
Atrial fibrillation, %	32.4	28.3	57.6	< 0.001	48.9	56.0	0.172
Coronary artery disease, %	10.2	9.9	12.5	0.266	12.6	12.6	1.000
Myocardial infarction (≤90 days), %	0.7	0.6	1.0	0.371	0.0	1.1	0.499
Prior aortic valve replacement, %	2.1	1.4	6.3	< 0.001	2.2	3.8	0.358
Prior CABG, %	3.9	3.5	6.3	0.070	7.1	5.5	0.518
Prior pacemaker, %	2.2	1.6	5.8	0.002	2.7	6.0	0.125
NYHA class III / IV, %	75.5	72.6	92.2	< 0.001	96.2	92.9	0.168
CCS class III / IV, %	4.1	3.9	5.2	0.381	5.5	5.5	1.000
Pulmonary hypertension, %	12.0	11.3	16.7	0.033	12.6	15.9	0.369
Emergency indication for surgery, %	3.9	4.3	1.6	0.070	1.1	1.6	1.000
Log EuroSCORE I, %	3.8 [1.9–9.0]	3.4 [1.6–7.8]	8.0 [4.0–14.3]	< 0.001	6.6 [3.3–14.3]	7.4 [3.9–12.4]	0.699

*Legend:* values represent percentage, mean ± SD or median [IQR]. *CABG* coronary artery bypass graft, *CCS* Canadian Cardiovascular Society, *COPD* chronic obstructive pulmonary disease, *Cr* creatinine, *IQR* interquartile range, *NYHA* New York Heart Association, *PAD* peripheral artery disease, *SD* standard deviation

In the PS-matched cohort, echocardiography revealed a largely comparable patient population in terms of MV pathology and further echocardiographic criteria (Table 2). There was a non-significant trend towards an increase in the left atrial diameter (56.3 vs. 53.7 mm; *p* = 0.091) and a significantly higher right atrial diameter (49.9% vs. 43.9%; *p* < 0.001) in the MVS + TVR group compared with the isolated MVS group.

**Table 2:** Mitral valve pathologies and echocardiographic parameters

	Isolated MVS (***N*** = 182)	MVS + TVR (***N*** = 182)	***P***-value
MV pathologies / echo parameters			
Degenerative MR, %	89.0	84.1	0.167
Functional MR, %	11.0	15.9	0.167
Acute endocarditis, %	3.3	3.8	0.778
Annulus dilatation, %	87.9	89.0	0.743
Annulus calcification, %	11.5	12.1	0.871
Mitral valve stenosis, %	8.2	9.3	0.711
Mitral valve insuff. grade ≥ II, %	99.5	98.4	0.623
General			
LVEF, %	51.8 ± 13.5	52.8 ± 12.7	0.486
LVEDD (mm)	55.5 ± 7.9	54.4 ± 8.8	0.248
LVESD (mm)	38.8 ± 9.6	37.1 ± 10.0	0.138
Left atrial diameter (mm)	53.7 ± 12.2	56.3 ± 12.9	0.091
Right atrial diameter (mm)	43.9 ± 11.3	49.9 ± 12.7	**< 0.001**

*Legend:* values are percentage or mean ± SD. *LVEDD* left ventricular end-diastolic pressure, *LVEF* left ventricular ejection fraction, *LVESD* left ventricular end-systolic pressure, *MV* mitral valve, *MVS* mitral valve surgery, *TVR* tricuspid valve repair

**Fig. 2:**
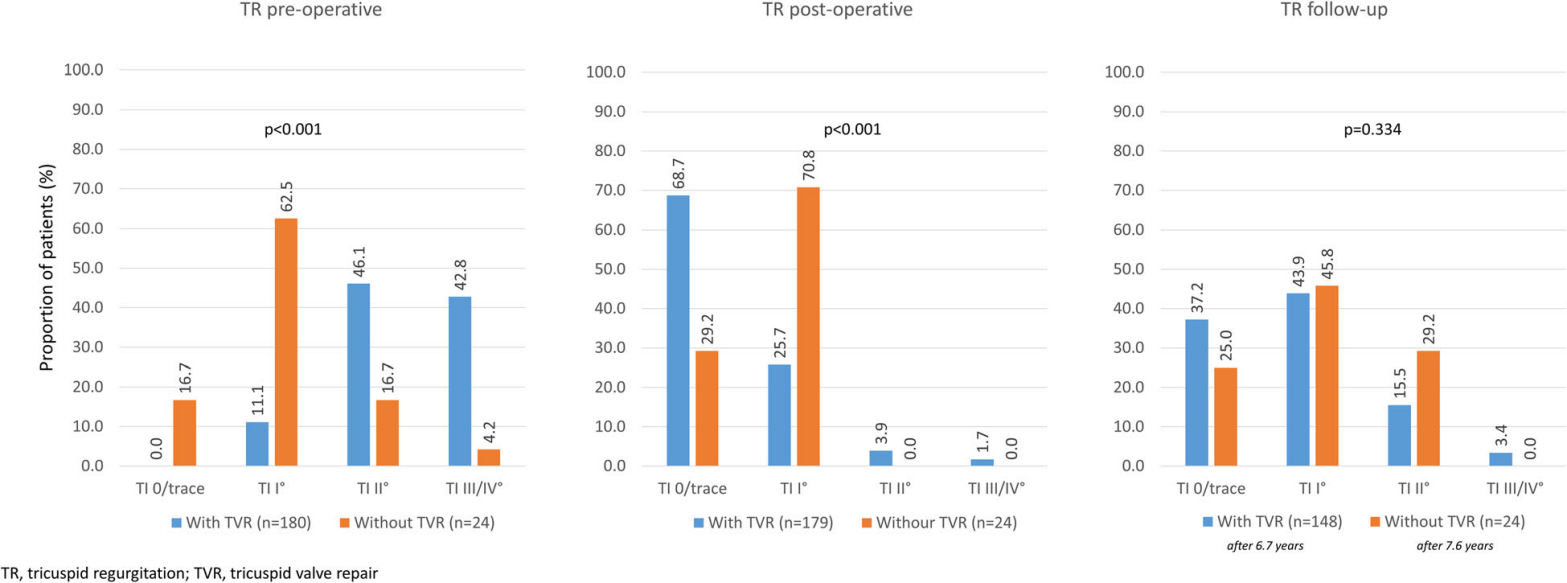
Tricuspid valve competency

Most patients (88.9%) in the MVS + TVR group had at least grade II tricuspid regurgitation (Table 3), while the majority of patients undergoing isolated MVS had either grade 0 or I regurgitation (79.2%) (Fig. 2, left panel) pointing at the principal reason for their consideration for MVS + TVR. Furthermore, MVS + TVR patients had increased right ventricular tricuspid annular plane systolic excursion (18.8 ± 3.9 mm) (Table 3); these data were not available for patients undergoing isolated MVR.

**Table 3:** Tricuspid valve-related parameters

	MVS + TVR (***N*** = 182) % or mean ± SD
Preoperative	
TV regurgitation ≥ grade II, %	88.9
RV TAPSE (mm)	18.8 ± 3.9
RVSP (mmHg)	52.9 ± 16.2
Vmax (cm/s)	316.6 ± 62.8
Intraoperative repair method	
De Vega Annuloplasty, %	1.1
Annulopasty ring, %	98.9
Cosgrove, %	12.5
CE classic, %	87.5
Annuloplasty ring size, %	
27 mm, %	0.6
28 mm, %	7.4
30 mm, %	26.7
32 mm, %	30.1
34 mm, %	29.0
36 mm, %	5.7
38 mm, %	0.6
Postoperative	
TV regurgitation ≥ grade II, %	5.6
Mean diastolic gradient (mm)	2.6 ± 1.7
RV TAPSE (mm)	16.9 ± 3.9
RVSP (mmHg)	57.6 ± 63.1
Vmax (cm/s)	272.5 ± 75.5
Follow-up	
TV regurgitation ≥ grade II, %	18.9
Mean diastolic gradient (mm)	2.7 ± 1.7
RV TAPSE (mm)	17.4 ± 3.4
RVSP (mmHg)	39.2 ± 15.3
Vmax (cm/s)	288.6 ± 83.8

*Legend:* values are percentage or mean ± SD. *MVS* mitral valve surgery, *RV* right ventricular, *RVSP* right ventricular systolic pressure, *SD* standard deviation, *TAPSE* tricuspid annular plane systolic excursion, *TV* tricuspid valve, *TVR* tricuspid valve repair, *Vmax* maximal velocity

### Procedural details and outcomes

The principal differences between the groups (Table 4) were higher duration of mechanical ventilation in the MVS + TVR group compared with the isolated MVS group (median 13 vs. 11 h; *p* < 0.001), as well as longer X-clamp time (90.5 vs. 66 min; *p* < 0.001), cardiopulmonary bypass time (136 vs. 95.5 min; *p* < 0.001), and overall procedure time (224 vs. 176 min; *p* < 0.001). Slightly longer ICU and hospital stays occurred in the MVS + TVR group, but did not reach statistical significance.

**Table 4:** Procedural details

	Isolated MVS (***N*** = 182)	MVS + TVR (***N*** = 182)	***P***-value
**Procedural details**			
Times			
Procedure time (min)	176.0 [155.0–203.8]	224.0 [190.8–261.3]	**< 0.001**
CPB time (min)	95.5 [80.0–125]	136.0 [110.0–173.0]	**< 0.001**
X-clamp time (min)	66.0 [55.0–82.0]	90.5 [66.8–108.3]	**< 0.001**
Duration mechanical ventilation (h)	11.0 [9.0–16.0]	13.0 [10.0–20.3]	**0.001**
Length of ICU (h)	24.5 [22.0–69.0]	27.0 [21.0–92.3]	0.650
Length of hospital stay (d)	11.0 [9.0–18.3]	13.0 [10.0–19.0]	0.087
MIC	24.2	30.8	0.159
Mitral valve repair			
AML repair	13.7	15.4	0.656
PML repair	49.5	35.2	**0.006**
Annuloplasty ring	80.8	80.8	1.000
Resection	37.4	21.4	**0.001**
Loops	15.4	17.6	0.572
Cleft plicature	9.9	10.4	0.862
Rate of successful repair ^a^	67.0	65.9	0.824
Mitral valve replacement			
Direct	19.2	18.7	0.894
MV replaced after failed repair	12.6	14.3	0.645
Biological	27.5	27.5	0.888
Mechanical	4.4	5.5	
Concomitant procedures			
Cryoablation	22.5	34.6	**0.011**
LAA closure	39.6	44.0	0.395
ASD closure	1.6	3.8	0.200
Myxom	0	0	n.a.

*Legend:* values are percentage or median [interquartile range]. ^a^Three patients were excluded as they died within 72 h after the intervention (electromechanical decoupling *n* = 1, low cardiac output and rhythm disturbances *n* = 1, cardiogenic shock and kidney failure *n* = 1)

*AML* anterior mitral valve leaflet, *ASD* atrial septal defect, *CPB* cardiopulmonary bypass, *ICU* intensive care unit, *LAA* left atrial appendage, *MV* mitral valve, *MVS* mitral valve surgery, *PML* posterior mitral valve leaflet, *TVR* tricuspid valve repair

There were no between-group differences with respect to the approach used for MV replacement. There were slight differences between the groups among those undergoing MV repair: posterior MV leaflet repair (49.5% vs. 35.2%; *p* = 0.006) and resection (37.4% vs. 21.4%; *p* = 0.001) were more common in patients undergoing isolated MV repair compared with those undergoing MV repair plus TVR. Concomitant procedures were more common in the MVS + TVR group, but only the difference in cryoablation reached statistical significance (34.6% vs. 22.5%; *p* = 0.011). Procedure-related complications differed slightly between the groups, but without statistical significance (Table 4).

### Functional outcomes

**Fig. 3:**
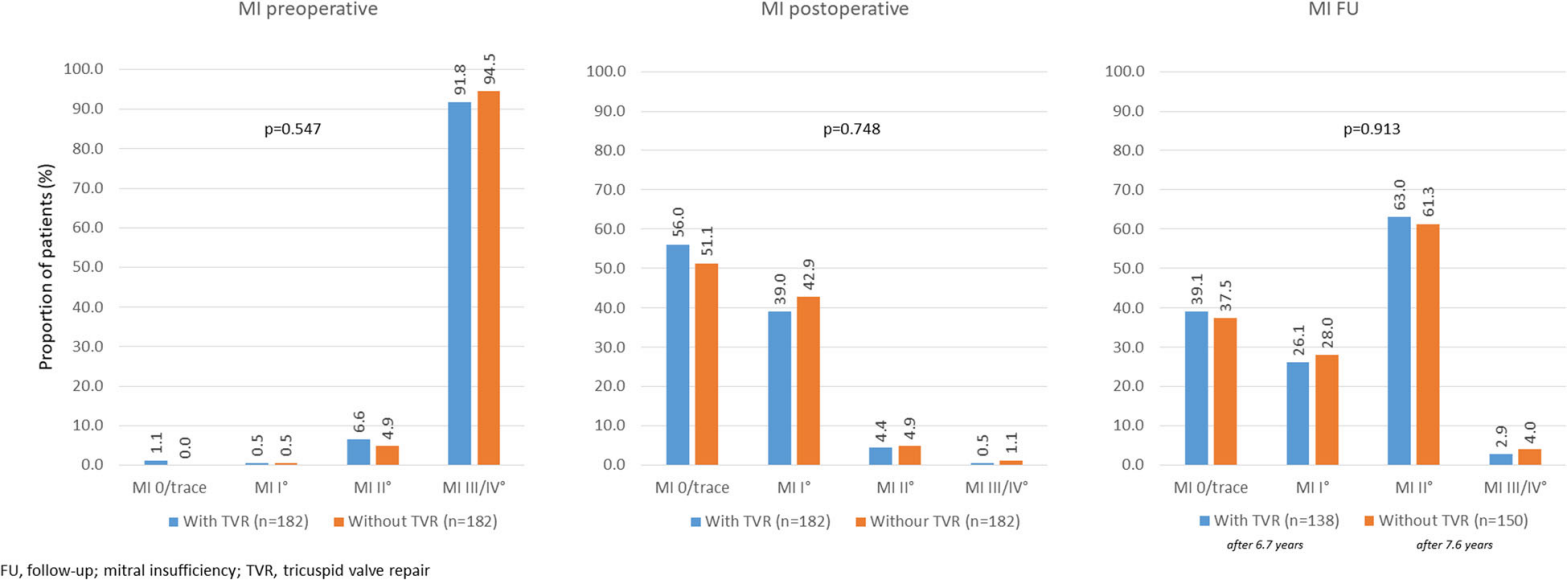
Mitral valve competency

Median MV gradients were similar in both groups post-surgery and remained so during long-term follow-up. While there was a substantial decrease in the proportion of patients with severe mitral insufficiency over time, differences between the groups were small and non-significant (Fig. 3).

Looking at the MVS + TVR group, there was a marked reduction in the rate of grade III/IV tricuspid insufficiency after the operation (from 42.8% before surgery to 1.7% postoperatively and 3.4% after long-term follow-up; Fig. 2). In patients not undergoing TVR, rates of tricuspid insufficiency were similar at baseline and after MVS. A slight deterioration was seen after long-term follow-up in either group.

**Fig. 4:**
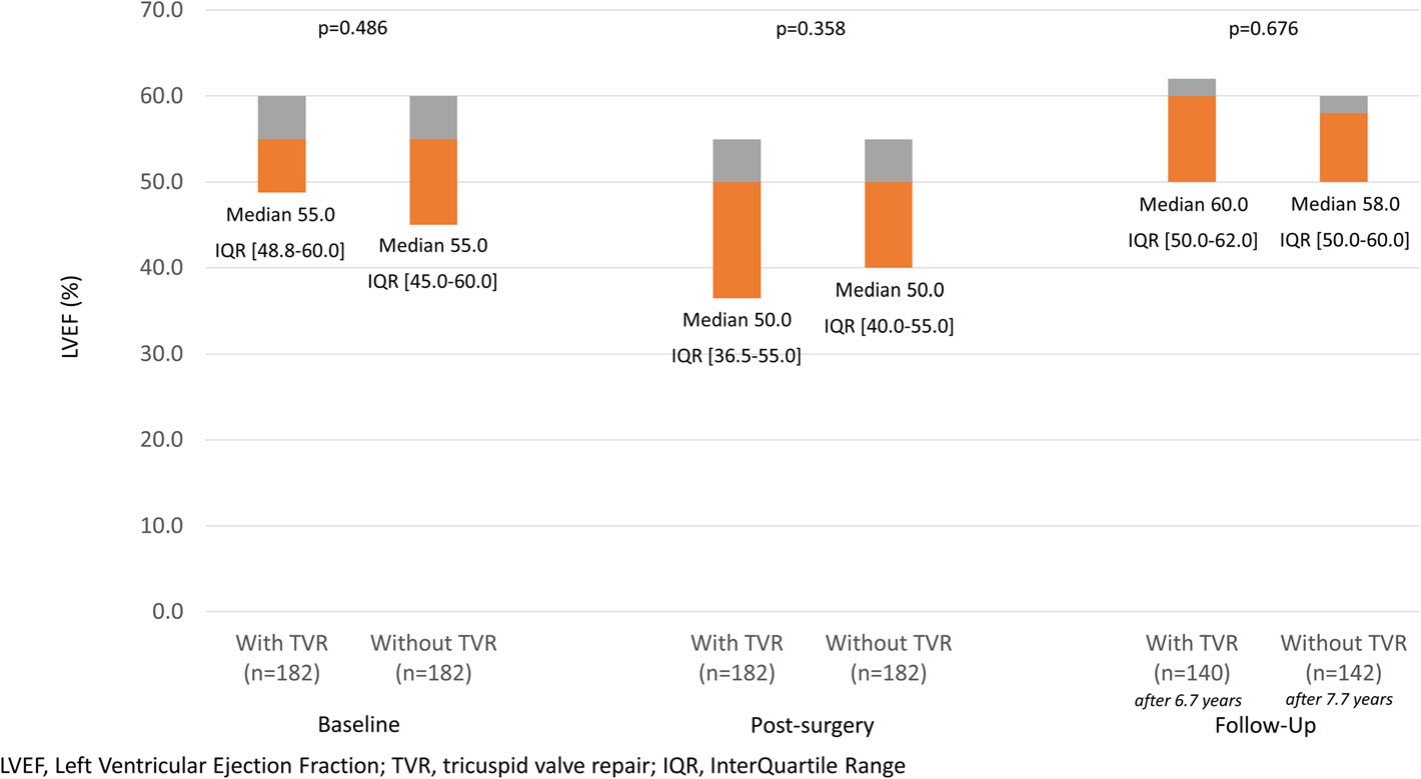
Left Ventricular Function

There was a temporary decline in LVEF immediately after the procedure in both groups (Fig. 4), which recovered during long-term follow-up. No differences were observed relating to the concomitant performance of TVR.

At baseline, most patients were in NYHA class III (81.3% MVS + TVR group and 86.8% isolated MVS group). After a mean follow-up of 7.2 years in the MVS + TVR group and 8.9 years in the isolated MVS group, most patients were in NYHA class I (50.4% MVS + TVR group and 48.2% isolated MVS group), with no significant difference in the distribution of classes between the MVS and MVS + TVR groups.

### Post-procedure clinical outcomes

There was no difference between the groups in terms of the rate of death within 30 days (Table 5). Implantation of a pacemaker was required more often after the combined procedure than after MVS (16.0% versus 8.8%; *p* = 0.037). There were no significant between-group differences with respect to rates of stroke, myocardial infarction or repeat MVS.

**Table 5:** Procedure-related complications and 30-day outcomes

	Isolated MVS (***N*** = 182)	MVS + TVR (***N*** = 182)	***P***-value
**Procedure-related complications**			
Postoperative mortality, %	1.6	1.1	1.000
Wound infection, %	3.3	3.3	1.000
Pericardial tamponade, %	6.6	8.2	0.548
AV block grade III, %	9.3	14.3	0.144
Pneumonia, %	8.2	11.0	0.374
Pneumothorax, %	0.5	0.5	1.000
Pleural effusion, %	3.3	6.0	0.214
Atrial Fibrillation, %	29.3	34.6	0.276
**30-day complications**			
Death, %	7.7	5.5	0.398
CV death, %	4.9	3.3	0.429
Non-CV death, %	2.7	2.2	1.000
Stroke, %	7.1	3.8	0.168
Acute renal failure, %	12.2	12.6	0.889
Myocardial infarction, %	0	0	n.a.
Pacemaker implantation, %	8.8	16.0	**0.037**
Repeat MV surgery, %	1.1	0.5	1.000

*Legend:* values are percentage or median [interquartile range]

*AV* atrioventricular, *CV* cardiovascular, *MV* mitral valve, *MVS* mitral valve surgery, *TVR* tricuspid valve repair

**Fig. 5:**
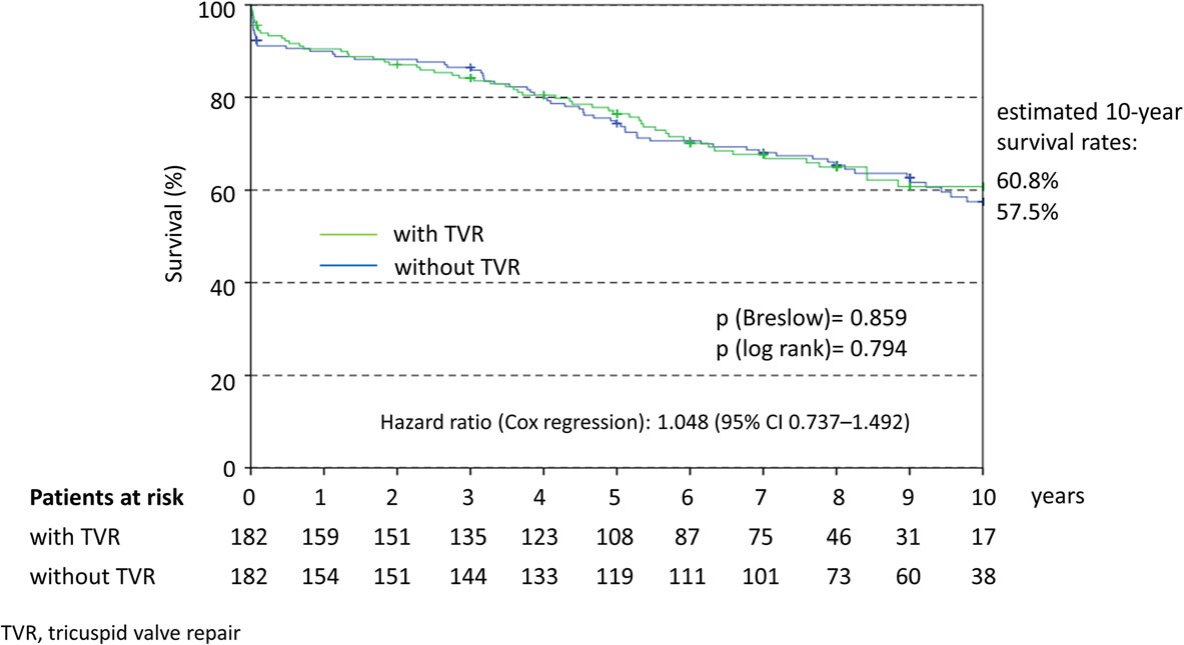
Kaplan Meier curve for long-term survival

Long-term survival is displayed in Fig. 5. The estimated 10-year survival rate was virtually identical for both groups (60.8% with MVS + TVR and 57.5% with isolated MVS; *p* = 0.794, log rank test) with an HR of 1.048 (95% confidence interval [CI] 0.737–1.492).

## Discussion

The main finding of this study is that long-term survival of MVS patients who undergo concomitant TVR because of moderate to severe TR is as good as the outcome of isolated MVS in patients with no or up to grade I TVR. There were no differences in short-term mortality or other complications, with the exception that PPI was required more often after the combined procedure.

We found no significant difference in either 30-day mortality or long-term (10-year) survival. This is consistent with a recent meta-analysis of studies comparing MVS with or without concomitant TVR, which found no difference in 30-day/in-hospital mortality (risk ratio 1.19, 95% CI 0.70–2.02; *p* = 0.52) or late mortality (incident rate ratio 0.87; 95% CI 0.63–1.24; *p* = 0.43) between the groups [6]. A comparable outcome was also noted in a meta-analysis that compared MVS with or without TVR specifically in patients who had preoperative mild-to-moderate TR [8]. The larger analysis by Tam et al. noted that there was a trend towards lower late mortality after concomitant TVR in randomized trials/adjusted studies (IRR 0.62, 95% CI 0.38–1.01; *p* = 0.06), but not in unadjusted studies [6]. Our study used PS matching adjusting for differences in patient characteristics, but not tricuspid valve parameters. The results are well aligned with most other PS-matched studies which also found no difference in survival between patients undergoing MVS with or without concomitant TVR [9–11], although one reported that the combined procedure produced better 5-year survival in patients with moderate-to-severe TR [12] and another found it reduced the risk of a combined endpoint of cardiac mortality/hospitalization for heart failure in patients with preoperative TR ≥2/4 [13]. The results should be interpreted with confidence as they would allow a liberal use of concomitant TV should disease characteristics mandate surgery.

One of the main rationales for performing concomitant TVR at the time of MVS is to prevent progression of TR and thus reduce the risk of a future need for reoperation to repair or replace the tricuspid valve [3, 6, 14, 15]. Moderate preoperative TR is a risk factor for severe postoperative TR in patients who do not undergo a concomitant TVR at the time of MVS. Repeat surgery for TR carries a high risk or morbidity and mortality [2, 4, 5]. The meta-analysis by Tam et al. confirmed that concomitant TVR at the time of MVS protected against future recurrent TR [6], and the meta-analysis of studies specifically involving patients with mild-to-moderate TR found that it led to a significantly higher rate of freedom from moderate-to-severe TR postoperatively [8]. Individual randomized trials and PS-matched analyses have reported reduced TR progression in patients treated with concomitant TVR [10, 13, 16, 17], including patients with no -more-than-mild TR at the time of surgery [9]. In our study, the severity of TR decreased markedly after concomitant MVS + TVR, and the rate of grade II or higher TR remained low during long-term follow-up. In the group that underwent MVS alone, the rate of grade II or higher TR increased slightly during long-term follow-up.

We noted no significant differences between the groups with respect to left ventricular functional outcomes or heart failure status either in the postoperative period or during long-term follow-up. This is consistent with the findings of randomized controlled trials [16, 17] and other studies which have also found that concomitant TVR alleviated heart failure symptoms [18]. We did not measure right ventricular parameters, but it has been shown previously that TVR at the time of MVS can reverse right ventricular remodelling and improve functional status, particularly in patients with annular dilatation [3, 16, 19].

Performing concomitant TVR at the time of MVS has implications for procedural times. We found a significant increase in the duration of mechanical ventilation, X-clamp and cardiopulmonary bypass times, which led to an increase in the overall procedure time of almost 50 min. The increase in cardiopulmonary bypass time (40.5 min) was somewhat greater than the mean difference reported in a meta-analysis (21 min), whereas the increase in X-clamp time (24.5 min) was similar to the mean value in the meta-analysis (21 min) [6]. We found no significant difference in length of ICU or hospital stay between the groups.

Even with the longer procedural time, TVR performed at the time of MVS is generally a safe procedure [2, 3, 6, 20]. We found no difference in procedure-related complications, and the only difference in 30-day complications was an increase in the need for PPI among patients who received the combined procedure. This has been reported previously [6, 21]. In the meta-analysis by Tam et al. the risk ratio for a new PPI in the group who underwent concomitant TVR was 2.73 (95% CI 2.57–2.89; *p* < 0.01) [6]. In most patients, this risk will generally be outweighed by the benefit that the combined procedure provides in terms of avoiding late TR.

### Limitations

This study had several limitations. 1) Patients in the current database had their surgery done in a long-time-window between 2005 and 2015 which allows a very long follow-up, but as surgical techniques develop and indications for concomitant TVR may change, this may result in potential bias that was not documented. 2) Furthermore the analysis does not allow to tell whether concomitant TVR in patients undergoing MVS should be performed irrespective of the degree of TR, but it reassures us to recommend TVR in patients with moderate to severe TVR as outcomes of the concomitant procedure are as as good as in those patients undergoing isolated MVR with none or trace TR. 3) There were no clear-cut and static criteria of when concomitant TVR was performed and surgeries were performed at the discretion of the treating surgeon. 4) Patients were documented from a large referral center where patients are referred to in complicated cases. As such we acknowledge the less than optimal outcome in some cases which we believe is due to this fact. 5) Non-randomized data analysis is potentially prone to bias. We matched two patients groups based on their patient characteristics at baseline to overcome this bias. On the other hand we advertently did not adjust for valve disease characteristics as they were the subject of investigation.

## Conclusions

The data show that the concomitant performance of TVR in patients undergoing MVS is a safe and effective procedure with good long-term outcomes. Patients can undergo MVS + TVR with confidence as it improves their prognosis up to the level of patients undergoing isolated MVS.

## Data Availability

Data are available from the corresponding author upon reasonable request.

## Abbreviations

AML: Anterior mitral valve leaflet
ASD: Atrial septal defect
AV: Atrioventricular
CABG: Coronary artery bypass graft
CCS: Canadian Cardiovascular Society
COPD: Chronic obstructive pulmonary disease
CPB: Cardiopulmonary bypass
Cr: Creatinine
CV: Cardiovascular
HR: Hazard Ratio
ICU: Intensive care unit
IQR: Interquartile range
LAA: Left atrial appendage
LVEDD: Left ventricular end-diastolic pressure
LVEF: Left ventricular ejection fraction
LVESD: Left ventricular end-systolic pressure
MV: Mitral valve
MVS: Mitral valve surgery
NYHA: New York Heart Association
PAD: Peripheral artery disease
PML: Posterior mitral valve leaflet
PPI: Permanent pacemaker implantation
PS: Propensity score
RV: Right ventricular
RVSP: Right ventricular systolic pressure
TAPSE: Tricuspid annular plane systolic excursion
TR: Tricuspid regurgitation
TVR: Tricuspid valve repair
TV: Tricuspid valve
Vmax: Maximal velocity
